# Antimalarial stocking decisions among medicine retailers in Ghana: implications for quality management and control of malaria

**DOI:** 10.1136/bmjgh-2023-013426

**Published:** 2021-09-21

**Authors:** Adams Osman, Fiifi Amoako Johnson, Simon Mariwah, Daniel Amoako-Sakyi, Samuel Asiedu Owusu, Martins Ekor, Heather Hamill, Kate Hampshire

**Affiliations:** 1 Department of Geography Education, University of Education, Winneba, Ghana; 2 Department of Population and Health, University of Cape Coast Faculty of Social Sciences, Cape Coast, Ghana; 3 Department of Geography and Regional Planning, University of Cape Coast Faculty of Social Sciences, Cape Coast, Ghana; 4 School of Medical Sciences, University of Cape Coast, Cape Coast, Ghana; 5 Directorate of Research, Innovation and Consultancy, University of Cape Coast, Cape Coast, Ghana; 6 Department of Sociology, Oxford University, Oxford, UK; 7 Department of Anthropology, Durham University, Durham, UK

**Keywords:** Health policy, Public Health, Malaria, Cross-sectional survey

## Abstract

Global health efforts such as malarial control require efficient pharmaceutical supply chains to ensure effective delivery of quality-assured medicines to those who need them. However, very little is currently known about decision-making processes within antimalarial supply chains and potential vulnerabilities to substandard and falsified medicines. Addressing this gap, we report on a study that investigated decision-making around the stocking of antimalarial products among private-sector medicine retailers in Ghana. Licensed retail pharmacies and over-the-counter (OTC) medicine retail outlets were sampled across six regions of Ghana using a two-stage stratified sampling procedure, with antimalarial medicines categorised as ‘expensive,’ ‘mid-range,’ and ‘cheaper,’ relative to other products in the shop. Retailers were asked about their motivations for choosing to stock particular products over others. The reasons were grouped into three categories: financial, reputation/experience and professional recommendation. Reputation/experience (76%, 95% CI 72.0% to 80.7%) were the drivers of antimalarial stocking decisions, followed by financial reasons (53.2%, 95% CI 48.1% to 58.3%) and recommendation by certified health professionals (24.7%, 95% CI 20.3% to 29.1%). Financial considerations were particularly influential in stocking decisions of cheaper medicines. Moreover, pharmacies and OTCs without a qualified pharmacist were significantly more likely to indicate financial reasons as a motivation for stocking decisions. No significant differences in stocking decisions were found by geographical location (zone and urban/rural) or outlet (pharmacy/OTC). These findings have implications for the management of antimalarial quality across supply chains in Ghana, with potentially important consequences for malaria control, particularly in lower-income areas where people rely on low-cost medication.

**WHAT IS ALREADY KNOWN ON THIS TOPIC**

- Malarial control requires efficient pharmaceutical supply chains to ensure effective delivery of quality-assured medicines to those who need them. However, very little is known about decision-making processes within antimalarial supply chains and potential vulnerabilities to substandard and falsified (SF) medicines.

**WHAT THIS STUDY ADDS**

- We confirm the uneven distribution of licensed medicine outlets across Ghana; rural areas and the less affluent north of Ghana have much poorer access to essential medicines.
- Private-sector medicine outlets are motivated to stock quality medicines rather than by purely financial considerations.
- Financial considerations were most important for the cheapest products and for outlets without a pharmacist.

**HOW THIS STUDY MIGHT AFFECT RESEARCH, PRACTICE OR POLICY**

- Private-sector outlets are crucial to the delivery of primary care to underserved communities.
- All medicine outlets must have trained staff to make informed stocking decisions.
- Ensuring access to affordable quality-assured antimalarials is key in both the fight against SF medicines and in moving towards genuine universal health coverage.

## Introduction

Well-governed pharmaceutical supply chain networks are integral to health systems and a critical enabler of universal health coverage (UHC), helping to ensure timely access to quality, safe and effective essential medicines. However, the proliferation of substandard and falsified (SF) medicines in low-income and middle-income countries (LMICs) suggests weaknesses within supply chains that threaten global health efforts such as malaria control and the progress towards UHC.^1–3^ Africa has the highest reported prevalence of poor-quality medicines, with antimalarials (19.1%) being the most affected drug class.^3^ Like any other African country, poor-quality medicines in Ghana are a major health threat, but the challenge has more to do with substandard than falsified products, where studies on the prevalence of substandard medicine products reported varied results. For example, Wilson *et al*^4^ and Tivura *et al*,^5^ respectively. found that 37% and 35% of antimalarials were substandard fueled by uncontrolled/managed borders, high drug demand, corruption, limited political will, poor coordination among institutions/countries, poverty, and illiteracy.^6^

We have observed that national and international efforts to curb the SF medicine menace largely focus on the public sector. However, in many LMICs, public, private and informal medicine distribution networks intertwine to form complex, fragmented and poorly regulated supply chains.^7 8^ Many of the world’s poor continue to source essential medicines from private-sector (including ‘informal’) outlets, owing to the inaccessibility of public facilities and frequent medicine stock-outs.^9^ Existing literature suggests that private-sector actors might sometimes behave (deliberately or otherwise) in ways that undermine supply chain robustness with potentially dire consequences for affordability, quality (SF issues) and availability of medicines.^10–14^ Indeed, there is now a significant body of evidence showing that stock-outs, cost and imbalances in demand and supply drive the emergence of SF medicines.^15 16^

This paper reports findings from a wider research project tracking decision-making through antimalarial supply chains in Ghana, from retail outlets up to manufacturers and importers. The focus here is on the retail outlets: the ‘last mile’ of medicine supply, whose decisions may directly impact on availability, quality, type, affordability of medicines to households and efficient flow of medicines to areas needed.^10–13^ Thus, the study sought to determine whether, in addition to quality considerations, retailers are influenced by other factors such as the financial cost of medicine and recommendations from health practitioners or suppliers. We predict that outlets in more economically constrained contexts will be more driven by financial imperatives and have less capacity to consider likely drug quality (based on outlets self-assessment of brand reputation, recommended efficacy by health specialists and personal experience rather than active ingredients), than those in more economically favourable contexts. More specifically, we hypothesise that:

- (H1) Outlets in more remote and poorer areas of the country (i.e., northern, rural) are more likely to be driven by financial considerations and less likely to be influenced by considerations of likely quality in antimalarial stocking decisions, compared with those located in more densely populated and wealthier areas of the country (i.e., southern/central, urban).
- (H2): Over-the-counters (OTCs) are more likely to be driven by financial considerations and less likely to be influenced by considerations of quality in antimalarial stocking decisions, compared with pharmacies because they are mostly in rural areas and low-income areas.
- (H3) For cheaper medicines, financial considerations are likely to be more important and quality less important in antimalarial stocking decisions, compared with more expensive medicines.

### Conceptual issues: antimalarial supply chain and SF medicines

After two decades of massive gains in the fight against malaria, recent data are warning of advances tapering-off. An estimated 14 million more cases and 47 000 more malaria deaths occurred in 2020 relative to 2019; an increase that persists even after adjusting for COVID-19-induced disruption of malaria services.^17^ Important global malaria strategy milestones for 2020 have been missed, and there is a risk of missing the 2030 target entirely if bold, immediate and smart actions are not taken to restore and improve gains.

National malaria control strategies for many endemic countries hinges on preventive chemotherapies such as intermittent preventive treatment of malaria in pregnancy, perennial malaria chemoprevention and seasonal malaria chemoprevention.^18 19^ Other preventive chemotherapies used depend on country-specific needs and may include post-discharge malaria chemoprevention for children at risk of malaria and recently treated for severe anaemia, and intermittent preventive treatment of malaria for school-aged children. The recommended medicines for these programmes rely on the pharmaceutical supply chains (sometimes including private sector actors) to reach the target populations. Thus, it is important for stakeholders to thoroughly understand the antimalarial supply chain specifically and safeguard it from vulnerabilities that promote the proliferation of SF antimalarials.

Ghana’s pharmaceutical supply chain resembles that of other LMICs in some ways but the local business environment, client preferences and policy initiatives such as the National Health Insurance Scheme (drugs are either subsidised or completely covered) are key distinguishing features. To date, very few studies have looked at the antimalarial supply chain in Ghana and none explores the relationship between various actors in the supply chain and the emergence of SF antimalarials.^20^ The ongoing threat of malaria and the limitations of current scholarship on antimalarial supply chains in Ghana suggests our study is timely and relevant in addressing a key threat to malaria control gains.

## Methods

### Study context

**Figure 1:**
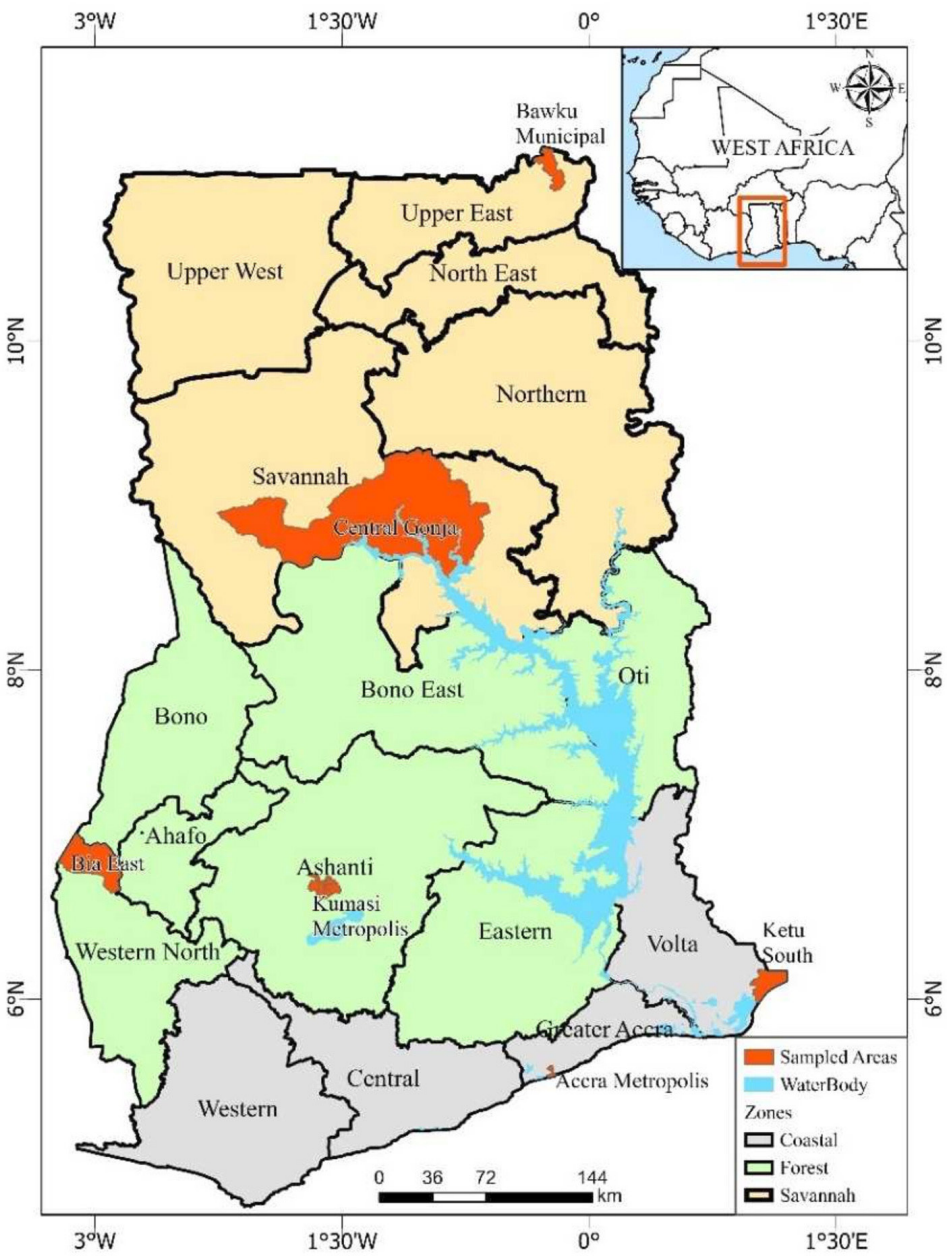
Sampled areas per regions in Ghana.

Ghana is a West Africa country divided into 16 administrative regions across 3 main agro-ecological zones (see figure 1), with a population of about 30.8 million.^21^ Ghana’s economy is classified as lower-middle income but with high multidimensional poverty (income, energy, food, formal education, health), persistent especially in rural areas and northern/savannah part of the country.^22 23^

Ghana’s climate (temperature: 24°C–32°C, rainfall: 780 mm–2160 m annually) and ecology (forest, dry equatorial, and savannah) make it highly susceptible to malaria and some other vector borne diseases. Malaria is responsible for over 38% of outpatient visits and 27.3% of health facility admissions,^24^ and for about 25% of under-5 deaths in Ghana.^25^ Ghana’s health system faces multiple challenges: limited healthcare facilities, uneven spatial distribution with concentration in the south (forest and coastal zones), limited health personnel, an inefficient health insurance scheme and high cost of medicines.^26 27^ Medicine distribution and sales are mainly privately owned, with limited quality control measures, which have implications for the proliferation of SF medicines in the country.^4^

### Study design

The data for the study are derived from a survey conducted in 2021 as part of the ‘STREAMS’ project (Strengthening private-sector Medicine Systems to tackle the persistence of poor-quality medicines in Africa), funded by the UK Medical Research Council. The research was codesigned by all authors at a workshop in Accra in March 2020. Data were collected from licensed retail pharmacies and OTC medicine retail outlets across Ghana to explore decision-making around the stocking of antimalarials. The study design was descriptive in nature, with the aim of describing the characteristics of sampled retail outlets and determining the factors influencing decisions to stock specific types and brands of antimalarials. Comparisons were made between outlets in different locations (border/non-border; rural/urban and across regions). Specifically, the study adopted a case series design, where a small sample of retail outlets were studied to help provide the basic understanding about stocking of antimalarials in Ghana (given the current paucity of information) and to provide data to inform future nationally representative studies.

### Sampling procedure

A two-stage stratified sampling design was adopted to ensure that the data collected were robust and guaranteed efficient use of resources. The sampling frame was the list of pharmacies (2879) and OTCs (12249) obtained from the Ghana Pharmacy Council as of 2019. At the first stage of the sampling process, the country was stratified into the three zones—Savannah/Northern (Savannah, Northern, North East, Upper East and Upper West regions), Forest/Middle (Western North, Ashanti, Ahafo, Eastern, Oti, Bono East and Bono regions) and Coastal/Southern (Western, Central, Greater Accra and Volta regions). These ecological zones are the statistical aggregation and reporting zones used by the Ghana Statistical Service.

At the second stage, the zones were stratified into border and non-border districts, because previous works in Africa and Ghana suggested that proximity to an international border (especially ‘leaky’ borders) promotes smuggling of substandard drugs.^5 28^ In each zone, one border and one non-border district were randomly sampled, ensuring that at least one district is as predominantly rural and the other urban (table 1).

**Table 1:** Number of registered and sampled pharmacies and OTCs

Zone	Border/non-border	Rural/urban	District	Number of registered			No of sampled		
				Pharmacies	OTCs	Total	Pharmacies	OTCs	Total
Savannah	Border	Urban	Bawku Municipal	5	43	48	5	15	20
	Non-border	Rural	Central Gonja	2	22	24	2	18	20
Forest	Border	Rural	Bia East	2	26	28	2	18	20
	Non-border	Urban	KMA	422	421	843	10	10	20
Coastal	Border	Rural	Ketu South	9	80	89	9	11	20
	Non-border	Urban	AMA	623	314	937	10	10	20
Total				1063	906	1969	38	82	120

AMA, Accra Metropolitan Area; KMA, Kumasi Metropolitan Area; OTC, Over The Counter.

Pharmacies and OTCs in each selected district were independently listed with unique identifiers. Using simple random sampling, 20 outlets (10 pharmacies and 10 OTCs) were selected from each district. In districts where the number of pharmacies were less than 10, all pharmacies were selected and supplemented with OTCs to achieve a total of 20 outlets.

### Data collection

A team of research assistants were deployed to each district to collect data from the sampled outlets. The position of each outlet was recorded using a handheld Global Positioning System (GPS) device, enabling superimposition of relevant secondary data. Data on the outlet (location, type, size, personnel, etc.) and antimalarial products (see below) were recorded using a computer-assisted personal interviewing tool (KoboToolBox), to minimise non-sampling errors.

### Outcome variables and covariates

The individual responsible for stocking decisions in each outlet was asked to list all the oral medicines currently in stock for treating acute malaria in adults, and then to identify three of their best-selling products: one more expensive, one relatively cheap, and one ‘mid-range’. (Note that these were relative assessments rather than absolute—that is, an ‘expensive’ product in one outlet might be a ‘cheap’ one in another). For each selected product, we recorded brand name, generic name, API, retail price, manufacturer, country of origin, expiry date and other details from the packages. Retailers were then asked to provide information on the source of each product (supplier, purchase price, quantity purchased and date) before explaining the reasons for choosing to stock that particular product. The latter question was asked in an open-ended way and respondents were encouraged to list all the considerations pertaining to each product. The interviewing tool was precoded with a series of possible responses that were identified from the pilot stage (plus an ‘other’ category”), but these were not shared with respondents, to avoid biasing responses.

To test the hypotheses above, we began by grouping the ‘reasons’ responses into two broad categories: financial and quality related. In practice, it can be very difficult to discern the quality of a medicine from visual inspection alone, so people often make use of proxies such as reputation, recommendation or personal experience.^29 30^ On this basis, we further subdivided the ‘quality’ responses into those based on personal experience or reputation (i.e., non-professional) and those based on recommendation from a ‘professional’ source. This resulted in three categories, operationalised as follows. Financial was coded ‘1’ if the respondent mentioned low price, profitability, or free delivery as considerations; otherwise, it was coded ‘0’. Experience/reputation was coded ‘1’ if the respondent mentioned customer demand, positive brand reputation, customers’ report of good previous experience, or personal (shop attendant) positive experience. Finally, professional recommendation was coded ‘1’ if the respondent mentioned recommendation by government, health professionals or suppliers; often prescribed by doctors; or public advertisement. In relation to the hypotheses above, the latter two categories (reputation/experience and professional recommendation) were used as a proxy for considerations of quality, as opposed to decisions driven primarily by economic motivations.

The primary covariates (ecological zone, rural–urban location, type of outlet and category of antimalarials) were selected based on the hypothesises of the study, while the confounders were selected based on the literature,^31^ and included border/non-border location, number of staff, average number of customers per day, years of operation and availability of qualified pharmacist. The confounders were included in the analysis to examine the true associative effects of the hypothesised (primary) variables.

### Data analysis

Frequency tables were used to describe the general characteristics of the sampled pharmacies and OTCs. Cross-tabulation was used to examine how the reasons for stocking vary by the primary and confounding factors, using χ^2^ tests to assess statistically significant differences. Binary logistic regression was employed to examine the primary and confounding factors that were statistically significantly associated with the reasons for stocking antimalarials. To prevent model overfit, only confounders significant at the 5% level were retained in the model.

### Patient and public involvement statement

Patients or the public were not involved in the design, or conduct, or reporting, or dissemination plans of our research.

## Results

### Background characteristics of the sampled outlets

Table 2 shows the background characteristics of the sampled outlets. Data were collected from 38 pharmacies (31.7%) and 82 OTCs (68.3%) located in rural (40.2%) and urban (60.8%) areas of Ghana. There were equal proportions (33.3%) of outlets in each of the three ecological zones (Southern, Middle, and Northern). Retail outlets were engaged in selling diverse antimalarials which were categorised into more expensive (38.7%), mid-range (34.4%) and cheaper (26.9%) based on cost. Based on the exchange rate at the time of fieldwork (US$1=GH¢7.6), the retail prices of the ‘more expensive’ medicines ranged from US$0.39 to US$8.16 (median US$1.97), while ‘mid-range’ products ranged from US$0.26 to US$3.42 (median US$1.05), and ‘cheaper’ drugs ranged from US$0.26 to US$1.97 (median US$0.66).

**Table 2:** Characteristics of sampled retail outlets

Characteristics	Frequency	Per cent
Type of outlet		
Pharmacy	38	31.7
OTC	82	68.3
Place of location		
Urban	73	60.8
Rural	47	39.2
Ecological zone		
Southern	40	33.3
Middle	40	33.3
Northern	40	33.3
Border/Non-Border		
Border	60	50
Non-Border	60	50
Price bracket (multiple response)		
Cheaper	100	26.9
Mid-range	128	34.4
More expensive	144	38.7
NO of staff		
One	73	60.8
Two	28	23.3
Three or more	19	15.8
NO of customers per day		
Less than 25	39	32.5
25–60	37	30.8
More than 60	44	36.7
Years of operation		
Less than 5 years	31	25.8
5–10 years	23	19.2
More than 10 years	66	55.0
Has qualified pharmacist		
Yes	119	50.7
No	25	49.3

OTC, over the counter.

### Bivariate analysis of the reasons for stocking antimalarials by outlet characteristics

As noted above, retailers’ decisions regarding which antimalarials to stock were categorised into financial, experience/reputation and professional recommendation (table 3). Overall, the most widely cited set of factors influencing decision-making were personal experience and reputation, mentioned in 76.3% of cases (95% CI 72.0% to 80.7%). This was followed by financial reasons (53.2% of cases, 95% CI 48.1% to 58.3%), and then professional recommendation (24.7% of cases, 95% CI 20.3% to 29.1%).

**Table 3:** Reported reasons for stocking antimalarials by pharmacies and OTCs

Background characteristics	Financial % (95% CI)	Experience/reputation % (95% CI)	Professional recommendation % (95% CI)
Overall	53.2 (48.1 to 58.3)	76.3 (72.0 to 80.7)	24.7 (20.3 to 29.1)
Primary factors			
Ecological zone	P value=0.456	P value=0.314	P value=0.047
Southern	48.7 (39.4 to 57.9)	81.4 (74.2 to 88.6)	16.8 (9.9 to 23.7)
Middle	56.7 (48.0 to 65.3)	74.0 (66.4 to 81.7)	26.0 (18.3 to 33.6)
Northern	53.8 (45.3 to 62.3)	74.2 (66.8 to 81.7)	30.3 (22.4 to 38.2)
Location	P value=0.123	P value=0.101	P value=0.062
Urban	50.4 (44.2 to 56.6)	73.8 (68.3 to 79.3)	21.8 (16.6 to 26.9)
Rural	58.9 (50.2 to 67.6)	81.5 (74.6 to 88.3)	30.6 (22.5 to 38.8)
Type of outlet	P value=0.208	P value=0.168	P value=0.611
Licensed pharmacy	49.4 (41.4 to 57.3)	72.7 (65.7 to 79.8)	23.4 (16.7 to 30.1)
Licensed OTC	56.0 (49.4 to 62.6)	78.9 (73.5 to 84.3)	25.7 (19.9 to 31.5)
Price bracket of antimalarial	P value=0.000	P value=0.314	P value=0.018
Cheaper	82.0 (74.4 to 89.6)	80.0 (72.1 to 87.9)	21.0 (13.0 to 29.0)
Mid-range	54.7 (46.0 to 63.3)	78.1 (70.9 to 85.3)	18.8 (12.0 to 25.5)
More expensive	31.9 (24.3 to 39.6)	72.2 (64.9 to 79.6)	32.6 (25.0 to 40.3)
Confounders			
Border/non-border location	P value=0.030	P value=0.201	P value=0.856
Border	58.8 (51.8 to 65.9)	79.1 (73.3 to 85.0)	25.1 (18.9 to 31.4)
Non border	47.6 (40.4 to 54.8)	73.5 (67.1 to 79.9)	24.3 (18.1 to 30.5)
No of staff	P value=0.364	P value=0.089	P value=0.100
One	54.0 (46.8 to 61.2)	79.7 (73.9 to 85.5)	21.9 (16.0 to 27.9)
Two	57.4 (47.4 to 67.5)	68.1 (58.6 to 77.6)	33.0 (23.4 to 42.5)
Three or more	47.3 (36.9 to 57.6)	78.0 (69.5 to 86.6)	22.0 (13.4 to 30.5)
No of customers per day	P value=0.650	P value=0.649	P value=0.278
Less than 25	54.2 (44.1 to 64.2)	74.0 (65.1 to 82.8)	27.1 (18.1 to 36.0)
25–60	56.2 (47.3 to 65.1)	76.0 (68.4 to 83.7)	28.1 (20.1 to 36.1)
More than 60	50.7 (42.7 to 58.6)	78.9 (72.4 to 85.5)	20.4 (14.0 to 26.8)
Year of operation	P value=0.450	P value=0.830	P value=0.537
Less than 5 years	50.0 (40.6 to 59.4)	76.4 (68.4 to 84.3)	23.6 (15.7 to 31.6)
5–10 years	50.5 (40.3 to 60.8)	78.5 (70.1 to 86.9)	29.0 (19.8 to 38.3)
More than 10 years	56.8 (49.3 to 64.3)	75.1 (68.6 to 81.7)	23.1 (16.7 to 29.4)
Has qualified pharmacists	P value=0.026	P value=0.275	P value=0.132
Yes	50.6 (45.1 to 56.2)	77.4 (72.8 to 82.1)	23.2 (18.5 to 27.9)
No	66.1 (54.3 to 78.0)	71.0 (59.6 to 82.4)	32.3 (20.5 to 44.0)

OTC, Over The Counter.

The analysis (table 3) revealed that financial reasons for stocking antimalarials were not dependent on ecological zone, rural/urban location, or type of outlet (pharmacy or OTC). However, price bracket did make a difference. For ‘cheaper’ products, financial reasons were mentioned in 82.0% of cases (95% CI 74.4% to 89.6%), compared with 54.7% (95% CI 46.0% to 63.3%) for ‘mid-range’ and 31.9% (95% CI 24.3% to 39.6%) for ‘more expensive’ products. Regarding the confounders, significant differences were found in mentions of financial considerations between border/non-border locations (p=0.03) and between outlets with/without a qualified pharmacist present (p=0.026). In outlets close to international borders, financial considerations were mentioned in 58.0% of cases (95% CI 51.8% to 65.9%), compared with 47.6% of cases (95% CI 40.4% to 54.8%) in non-border areas. Further, 66.1% (95% CI 54.4% to 78.0%) of outlets without a pharmacist present cited financial reasons for stocking decisions, compared with 50.6% (95% CI 45.1% to 56.2%) of those with a pharmacist present.

No significant differences in the proportions citing experience/reputation were found in either the primary factors (ecological zone, rural–urban locatio, and outlet type or price bracket) or any of the confounders. By contrast, ‘professional recommendation’ was significantly more likely to be mentioned in relation to ‘more expensive’ antimalarials 32.6% (95% CI 25.0% to 40.3%), compared with products that were ‘mid-range’ (18.8% of cases, 95% CI 12.0% to 40.3%) or ‘cheaper’ 21.0% of cases, 95% CI 13.0% to 29.0%). ‘Professional recommendation’ was also more likely to be cited as a driver of decision-making in the Northern part of Ghana (30.3% of cases, 95% CI 22.4% to 38.2%) and in the Middle Belt (26.0% of cases, 95% CI 18.3% to 33.6%), compared with the Southern zone (16.8% of cases, 95% CI 9.9% to 23.7%).

### Multivariate analysis of factors associated with antimalarial stocking decisions

Table 4 shows the primary factors hypothesised to be associated antimalarial decisions and confounding factors found to be statistically important (p<0.05) in the bivariate analyses above. Although the direction of effects was hypothesised for financial motivations, this only reached statistical significance for one of the primary factors (price bracket) and one of the confounders (presence of a pharmacist). Financial considerations were significantly more likely to factor into decision-making for cheaper products, followed by mid-range, and least likely to matter for more expensive medicines (p<0.01). Presence of a pharmacist was found to be associated with a lower likelihood of financially driven stocking decisions compared with outlets with no pharmacist present (p<0.05). None of the hypothesised and confounding factors were associated with either personal experience/reputation or professional recommendation in the multivariate analysis.

**Table 4:** Factors associated with reasons for stocking antimalarials

	Financial OR (95% CI)	Experience/reputation OR (95% CI)	Professional recommendation OR (95% CI)
Ecological zone			
Southern	1.00	1.00	1.00
Middle	1.45 (0.82 to 2.58)	0.60 (0.32 to 1.12)	1.67 (0.88 to 3.18)
Northern	1.06 (0.57 to 1.96)	0.55 (0.28 to 1.06)	1.89 (0.97 to 3.68)
Location			
Urban	1.00	1.00	1.00
Rural	1.39 (0.78 to 2.46)	1.69 (0.9 to 3.2)	1.38 (0.76 to 2.5)
Type of outlet			
Licensed pharmacy	1.00	1.00	1.00
Licensed OTC	1.14 (0.67 to 1.94)	1.19 (0.69 to 2.05)	0.92 (0.52 to 1.6)
Price bracket			
Cheaper	1.00	1.00	1.00
Mid-range	0.22 (0.11 to 0.42)**	0.85 (0.44 to 1.64)	0.84 (0.43 to 1.63)
More expensive	0.08 (0.04 to 0.15)**	0.63 (0.33 to 1.17)	1.72 (0.94 to 3.15)
Confounders			
Has qualified pharmacists			
Yes	1.00		
No	1.99 (1.02 to 3.87)*		

*p<0.01, **p<0.05.

OTC, over the counter.

Disaggregated analysis (online supplemental appendix 1) revealed that profitability considerations were significantly more likely to factor into decision-making for outlets in rural areas (p<0.01), and border outlets likely to consider low price antimalarial (p<0.05). Personal positive experience was significantly likely to be considered in antimalarial stocking decisions in the rural areas. Customer previous experience was likely considered in the northern ecological zone but positive brand less likely in the same ecological zone.

10.1136/bmjgh-2023-013426.supp1 Supplementary data

## Discussion

Private-sector pharmaceutical supply chains are integral to the fight against SF and the success of global health initiatives like the attainment of UHC and malaria control milestones. Cognisant of the complex nature of private sector pharmaceutical supply chains in LMICs and the vital role that medicine retail outlets play in ensuring that good quality antimalarials get to patient,^32^ this study investigated the reasons behind antimalarial stocking decisions at the medicine retail outlet level in a country with a fragile health system and suboptimal regulatory structures (although better than many other countries in the Region).

The emergence of personal experience and reputation (one group of proxies for quality) as the overall leading set of motivations driving antimalarial stocking decisions in this study is quite instructive. Although international stakeholders are gradually warming up to the reality that the private sector play a vital role in malaria control, there is still some amount of scepticism about the financial motivations of the sector.^33 34^ Such scepticism is perhaps fueled by the overly commercial orientation of private-sector retail outlets and studies suggesting that pricing and profitability are the main drivers of stocking practices of retail outlets.^35^ Thus, our finding that perceived quality is apparently more important overall than financial considerations in antimalarial stocking decisions in Ghana is refreshing. However, financial motivations did emerge as the second most popular driver of stocking decisions overall.

In considering variation between different types/locations of outlets and products, the rejection of our hypothesis (H1) that outlets in poorer and more remote areas are likely to prioritise profit over quality of antimalarials is noteworthy. Despite a palpable rural–urban dichotomy in Ghana and substantial socioeconomic differences between zones and region,^29^ outlets in the rural and more northern areas appeared not to be any more motivated by financial considerations than their counterparts in relatively affluent areas. This finding is intriguing considering the dire economic situation in rural parts of Ghana, with outlets operating on small margins and many people unable to afford anything but the cheapest medicines, where one might expect that economic imperatives would be particularly pressing. Other work suggests that outlets in rural areas are often operated by persons from those communities who are highly trusted and widely known, and thus may be particularly motivated to ensure their clients receive effective treatment wherever possible.^36–38^

The impact of financial considerations on stocking decisions came to the fore in two other situations: price bracket and presence/absence of a pharmacist. However, H2 was not accepted as there were no significant differences in financial consideration stocking decisions between OTCs and pharmacies. Outlets’ financial considerations were influenced stocking decisions for ‘cheaper’ compared with ‘more expensive’ antimalarials supporting H3 (For cheaper medicines, financial considerations are likely to be more important and quality). This is perhaps unsurprising: lower-cost medicines tend to be purchased by the poorest within a community, for whom pricing is likely to be particularly sensitive, thereby incentivising retailers to select the very cheapest products. In other words, it may be that selecting primarily on the basis of perceived quality is a ‘luxury’ than can only be entertained in less economically constrained circumstances. The Global Fund’s Affordable Medicines Facility for Malaria (AMFm) project, which provided a subsidy for the purchase of ACTs (artemisinin combination therapies, currently recommended by the WHO for first-line treatment of acute malaria) was a direct response to meet the demand for efficacious, low-cost in LMICs.^39^ Evidence from Ghana suggests that the AMFm was effective in making Quality-Assured ACTs readily available, even in rural medicine outlets.^40^ Similarly, government subsidies were reported to have improved access to antimalarials and reduced SF medicines by 50% in rural Uganda.^41^ Our finding that financial considerations still drive the stocking of lower-cost antimalarials suggests that self-sustaining initiatives fashioned after AMFm might be important in curbing the SF medicine threat.

Second, the presence of a qualified pharmacist was associated with a lower influence of financial considerations in stocking decisions, compared with outlets without a pharmacist. Other studies have suggested that the unavailability of trained pharmacists at outlets promotes ‘irrational’ dispensing but very little is known about the impact on stocking decisions. If the person responsible for stocking decisions lacks the necessary pharmacological knowledge, they may rely more on a pricing heuristic. This is important because pharmacists are more likely to be present in large, urban, upscale pharmacies than in small, rural, OTC outlets.

By contrast, none of our hypotheses regarding the impact of outlet location (zone or rural/urban) or outlet type (pharmacy/OTC) on antimalarial stocking decisions were supported. Some previous studies in LMICs^42^ have reported differences between the two, claiming that pharmacies are more loyal to products and rent-seeking, and are thus more likely to stock products based on their personal interest. However, such tendencies were not evident in our study. It may be that the sample sizes used in our study were too small and lacked sufficient statistical power (in most cases, the direction of the effect was as predicted but did not reach statistical significance), or it may be that there really are no differences in these respects. As noted above, it would be useful in the future to scale-up this study to be nationally representative.

## Conclusions

Several findings from this study have practical implications for SF medicines and malaria control in Ghana. First, through the sampling, our study confirms the uneven distribution of licensed medicine outlets across Ghana, with those living in urban areas and in the relatively affluent southern parts of the country having much better access (especially to pharmacies) than those living in more rural areas and in the less affluent north. While this is not a new finding, it continues to affect access to essential medicines like antimalarials for large parts of the population. Second, broadly speaking, private-sector medicine outlets across the country were stocking antimalarials based on the price bracket of the drug. It is clearly time that policy-makers recognised the reality that private-sector outlets perform a crucial role in delivering primary care to underserved communities, often doing the best they can be in difficult circumstances, and work to support their efforts.

Third, in two specific sets of circumstances, financial considerations came more to the fore: for the cheapest products and for outlets without a pharmacist present. These cases present potential supply-chain vulnerabilities, where price may trump considerations of quality. It is notable that both are more likely to affect disadvantaged rural communities, who are less likely to be able to afford anything but the lowest-cost medicines and who may be less likely to have access to an outlet staffed by a qualified pharmacist. Ensuring that all medicine outlets have staff with adequate training to make informed stocking decisions (whether or not they are fully qualified pharmacists) and ensuring that access to affordable quality-assured antimalarials continues in a sustainable manner in the post-AMFm era are therefore key priorities in both the fight against SF medicines and in moving towards genuine UHC.

10.1136/bmjgh-2023-013426.supp2 Supplementary data

## Data Availability

Data are available on reasonable request.
