# Supplementary material for: Antimalarial stocking decisions among medicine retailers in Ghana: implications for quality management and control of malaria
Source: BMJ Glob Health. 2021 Sep 21;6(Suppl 3):e013426. doi: 10.1136/bmjgh-2023-013426 (PMC10846845; doi:10.1136/bmjgh-2023-013426)
Supplement: Supplementary data [file bmjgh-2023-013426supp001.pdf]

Appendix 1: Multivariate analysis of outlet location and stocking decision of antimalarials

	Financial reasons		Professional recommended	Experience/reputation		
Variables	Profitable OR [95% CI]	Low price OR [95% CI]	Often prescribed by doctors OR [95% CI]	Personal positive experience OR [95% CI]	Customers previous experience OR [95% CI]	Positive brand reputation OR [95% CI]
Place of location						
Urban	0.43 [0.27, 0.69]**			0.54 [0.30, 0.94]*		
Rural	1			1		
Ecological zone						
Southern					0.47 [0.27, 0.83]**	2.44 [1.38, 4.30]**
Middle					0.81 [0.49, 1.35]	1.46 [0.83, 2.57]
Northern					1	1
Border location						
Border		1.73 [1.13, 2.65]*		2.01 [1.12, 3.58]*		0.58 [0.37, 0.92]*
Non-border		1		1		1
Has qualified pharmacist						
Yes			0.41 [.021, 0.80]*			
No			1			
