# Supplementary material for: Antimalarial stocking decisions among medicine retailers in Ghana: implications for quality management and control of malaria
Source: BMJ Glob Health. 2021 Sep 21;6(Suppl 3):e013426. doi: 10.1136/bmjgh-2023-013426 (PMC10846845; doi:10.1136/bmjgh-2023-013426)
Supplement: Supplementary data [file bmjgh-2023-013426supp002.pdf]

Appendix S1 – Reflexivity Statement

1. How does this study address local research and policy priorities?

The findings of this study have implications for the management of anti-malarial quality across supply chains in Ghana, with potentially important consequences for malaria control, particular in lower-income areas where people rely on low-cost medication.

2. How were local researchers involved in study design?

The first category of local researchers involved were those with extensive experience of conducting, leading, or organising international research collaborations (SM, DAS and SAO in Ghana, Tanzania, and Malawi), FAJ in Ghana and UK, and ME in Ghana and Nigeria. The second category were local researchers for whom this was their first international collaboration (AO). In addition, there were high-income country researchers with extensive experience of conducting, leading, or organising international research collaborations involving low- and middle-income countries (KH and HH). Most of the authors originate from, and reside, in Ghana. The local researchers (AO, FAJ, SM, DAS, SAO, and EM) were involved in the design of the project, and they led the data collection and manuscript preparation.

3. How has funding been used to support the local research team?

This project started with a workshop attended by all authors in which the research was co-designed. Funding has also been used to support the local research team to employ and train research assistants in data collection and collation and to train colleagues in the University of Cape Coast (UCC), Ghana in several data analysis techniques such as GIS. These capacity building activities will continue to be supported by the senior authorship team to develop.

4. How are research staff who conducted data collection acknowledged?

All local research staff who attended the research design workshop were included as authors. Each team member was designated and delivered specific role(s) during the writing process (see acknowledgements).

5. Do all members of the research partnership have access to study data?

All members of the partnership have access to data.

6. How was data used to develop analytical skills within the partnership?

Attendees of the research workshop contributed directly to the research design. Fortnightly meetings where subsequently held via MS Teams over a 2-year period with the full research team to discuss progress and co-develop solutions for any problems that arose in the conduct of the research. The analytical skills of the local research team have been enhanced through constant feedback on project reports and draft manuscripts.

7. How have research partners collaborated in interpreting study data?

During the fortnightly MS Teams meetings, hypotheses and data analysis strategies were discussed in a structured format. Short papers and presentations were given by members of the team presenting the findings, which were discussed by the whole team. These frequent meetings have led to the preparation of this manuscript.

8. How were research partners supported to develop writing skills?

The authors are composed of senior and early career academics. The early career researchers (AO) on the authorship team were supported by senior colleagues to develop and refine their writing skills. This was achieved through the constant feedback on draft manuscripts by senior colleagues.

9. How will research products be shared to address local needs?

This article will be published as open access and findings will be discussed with research and policy leaders in global health in Ghana.

10. How is the leadership, contribution, and ownership of this work by LMIC researchers recognised within the authorship?

Authors AO, FAJ, SM, DAS, SAO, and ME worked as part of the senior authorship team in developing this manuscript, and their contribution has been recognised by the author order.

11. **How have early career researchers across the partnership been included within the authorship team?**

AO is an early career researcher and is first author.

12. How has gender balance been addressed within the authorship?

Six authors are male (AM, FAM, SM, SAS, SAO, and ME) and two authors female (HH and KH)

13. How has the project contributed to training of LMIC researchers?

The authorship team is primarily composed of senior researchers apart from AO who has been supported in his writing and analytical skills as first author.

14. What safeguarding procedures were used to protect local study participants and researchers?

Safeguarding issues were directly addressed through the ethics approval given by the IRBs at UCC Ghana and Durham University UK.
